# Coding Dancing Figural Animations: Mathematical Meaning-Making Through Transitions Within and Beyond a Digital Resource

**DOI:** 10.1007/s40751-022-00118-x

**Published:** 2022-11-23

**Authors:** Chronis Kynigos, Myrto Karavakou

**Affiliations:** 1 Educational Technology Lab, National Kapodistrian University of Athens, Athens, Greece; 2 Linnaeus University, Vaxjo, Sweden

**Keywords:** Digital technology, Transitions, Mathematics, Art, UDGS, Mathematical meaning-making

## Abstract

We investigate three 8th-grade students’ mathematical meanings developed in the context of using linked representations to generate animations of figural models tuned in musical rhythm in “MaLT2,” a programmable Turtle Geometry in 3D resource affording dynamic manipulation of variable values. We adopted a modified version of the UDGS (Using, Discriminating, Generalizing, Synthesizing) model, introduced by Hoyles and Noss in 1987, in order to frame and analyze students’ mathematical meaning-making process involving setting out goals; posing conjectures; using mathematical ideas to test them; and exploring, generalizing, and expanding these ideas. This dynamic process was contextualized and connected to a flow of two different types of transitions: (1) transitions within the different representations of MaLT2 and (2) transitions beyond MaLT2, among the representational contexts of the digital microworld, artistic ideas, and abstract mathematics. In our analysis, we use this theoretical concept to trace the kind of mathematical meanings connected to multidisciplinary notions embedded in dance and music, such as synchronicity, symmetry, periodicity, and harmony, emerging from this learning context. We also look into the way these mathematical meanings were gradually evolved from being implicitly integrated in digital and artistic ideas to being reflected on and generalized.

In this article, we discuss 8th-grade (aged 14–15) students’ learning while they were engaged in an activity involving open artistic creations with a digital resource. We studied the way their mathematical meanings evolved through a flow of transitions at two levels: (a) among different representational contexts of this specific resource which integrates Logo programming, 3D Turtle Geometry, and dynamic manipulation of variable values; (b) among digital, artistic, and mathematical representations of ideas around music and dance. To do this, we found ourselves needing to disengage from assumptions inherent in some pre-virtual cultures regarding stagnant curriculum structures, lack of student agency, and the process of mathematical meaning-making.

The emergence of digital technology in education has enabled and cultivated new epistemological and pedagogical perspectives on learning and doing mathematics. It has opened up empowering possibilities for students to engage with mathematical thinking and construct mathematical meanings (diSessa, 2018; Drijvers et al., 2010; Kynigos, 2015). Kaput (1999) has argued that digital technology heralded a new kind of culture in education—the “virtual culture”—that provides qualitatively different affordances than former kinds. This novel culture brought out new representational forms and allowed students to engage in creative approaches within abstract mathematical situations by incorporating socio-cultural contexts (Kaput, 1989; Kynigos & Diamantidis, 2021; Shaffer & Kaput, 1998). diSessa (2018) discusses the way computers could fundamentally change perceiving and learning of mathematics by means of a profoundly influential “computational literacy” that would outweigh textual literacies in the near future.

Nevertheless, as supported by relevant reviews, the wave of transformation raised by this new virtual computational paradigm in education has been confronted with a platonic vision of mathematical knowledge, which is strictly organized in the stabilized corpus of school curriculum (Bray & Tangney, 2017; Forsström & Kaufmann, 2018; Hegedus & Moreno-Armella, 2014; Hoyles & Noss, 2003; Kynigos, 2019). This confrontational situation regarding the challenges posed by the virtual culture led us to adopt an approach allowing considerations for restructurations of established mathematical curriculum infrastructures and a deliberate reconsideration of learning and teaching possibilities.

Hence, we recognize the need to investigate the nature of mathematics emerging from students’ own use of digital technology, without taking curriculum infrastructures for granted. We consider the significance of the adaptation of curriculum structures to data emerging from this new transformative wave and not the other way around (Hoyles et al., 2020; Wilensky & Papert, 2010). Designing activities for students should focus on fostering a progressive intercourse between the digital medium and the learner, embracing new ways of using, and expressing mathematics (Hegedus & Moreno-Armella, 2014).

For this study, we framed this type of intercourse between students and a digital resource, in terms of transitioning between different representations of mathematized situations. The components of music and dance were integrated into our design for various reasons. There is a wide range of existing research that supports the pedagogical affordances of embedding musical concepts such as rhythm, harmony, melody, and tempo—which own a deep mathematical status while providing means of application for abstract mathematical objects—into mathematical learning contexts (Bamberger, 2013; Bamberger & diSessa, 2003; Courey et al., 2012; da Silva, 2020). However, studies focusing on the potentiality of the combination of digital resources and artistic contexts in mathematics education remain limited. We suggest that this integration is a fertile ground for investigating students’ mathematical meaning-making process. In addition, including music and dance aimed at provoking the transitioning processes and, at the same time, bringing out an aspect of human culture from within mathematics. In this way, the system of representations would be extended by linkages between abstract mathematical concepts and an external practical-artistic context closer to students’ personal sensibilities. Finally, we developed a theoretical construct, inextricably linked with the digital resource, that we based on the UDGS model (Hoyles & Noss, 1987). Our goal was to capture the potential impact of transitioning among different representational contexts on students’ meaning-making process as well as to investigate the type of their mathematical meanings shaped within this learning context.

## Theoretical Background

### Building on Constructionist Theoretical Constructs: Revisiting the UDGS Model

The theoretical foundation of this study relies on some long-standing ideas originating from the pedagogical movement of constructionism (Kynigos, 2015; Papert & Harel, 1991). The main theoretical concepts that we adopted have their roots back in the 1980s and 1990s, when Richard Noss and Celia Hoyles made some first attempts to conceptualize and describe students’ mathematical meaning-making processes while using expressive computational tools, such as Logo programming (Hoyles & Noss, 1987, 1992; Noss & Hoyles, 1996).

They were based on the key principle of constructionism, according to which students’ mathematical ideas are both shared and progressively shaped while interacting with technological tools in order to construct or tinker digital artefacts within a “microworld” (Hoyles & Noss, 1992; Kynigos, 2007; Papert, 1980). The concept of microworld was introduced by Papert as a self-contained computational world where students can “learn to transfer habits of exploration from their personal lives to the formal domain of scientific construction” (Papert, 1980, p. 177). Microworlds were later on described as “computational environments embedding a coherent set of scientific concepts and relations designed so that, with an appropriate set of tasks and pedagogy, students can engage in exploration and construction activity rich in the generation of meaning” (Kynigos, 2007, p. 337). Due to their highly editable nature, microworlds can provide viewable and analyzable links between students’ interactions within them and their meanings of mathematical concepts in use.

**Fig. 1:**
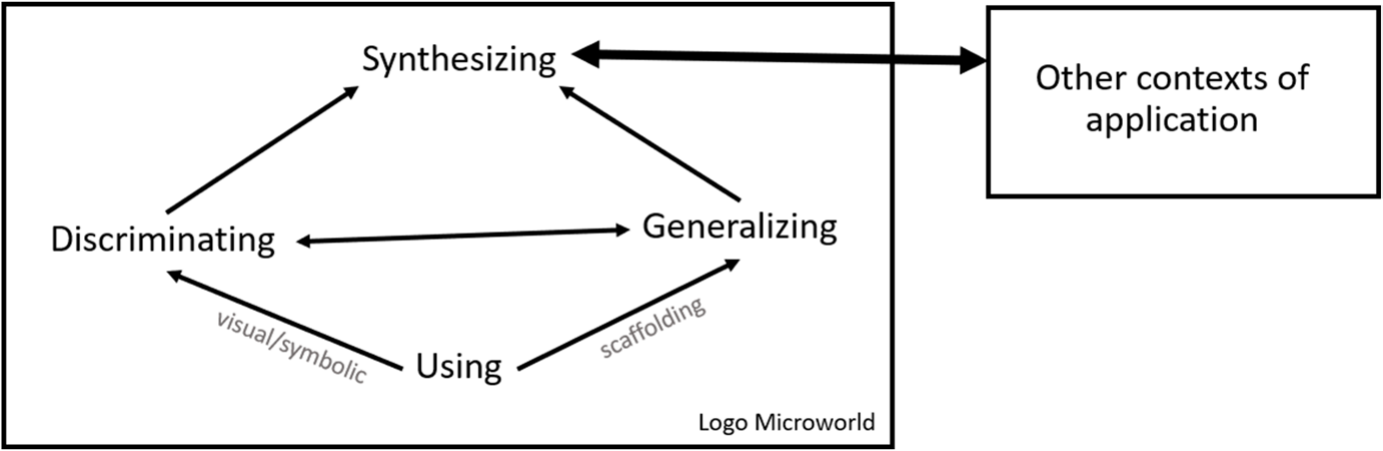
The UDGS model as presented by Hoyles and Noss (1987)

In 1987, Noss and Hoyles introduced the UDGS theoretical model and provided an articulated way to frame the progressive development of students’ mathematical meanings in terms of their activity within a microworld. This model was used for conceptualizing the phases of mathematical meaning-making, starting from an empirical, intuitive level and progressively evolving to conscious appreciation of generalized relationships among the mathematical concepts in use along with the digital tools (Hoyles & Noss, 1992; Kynigos, 2015; Laborde et al., 2006). In this framework, a mathematical meaning is the way that a student uses and thinks of a certain mathematical concept. The UDGS model (Fig. 1) involves the following dynamically related components of:

- Using: where mathematical concepts are used—without much attention to their actual meaning—as tools for functional purposes to achieve particular goals.
- Discriminating: where the different parts/elements of mathematics used as a tool are progressively distinguished and become explicit.
- Generalizing: where mathematical patterns in properties or relations of the tools are consciously extended and expressed.
- Synthesizing: where the generalized ideas used in the tools are consciously integrated with other contexts of application or representation—including pure mathematical ones (e.g., algebraic expression in paper-and-pencil).

According to Hoyles and Noss (1987), students’ meaning-making process can be mapped to moving towards all phases of the UDGS model through a circle of conjecturing, testing, and debugging actions. They start from an empirical, instinctive mode of activity, where mathematical concepts are implicitly used and transferring to a reflective one. This shift in the activity modes is signified by progressively discriminating the mathematical relations and concepts underpinning the behavior of the tools and generalizing them locally to the situations from which they emerged.

At the final phase of this model, students get to synthesize their generalizations in different representational registers outside the specific technology used, which can be either different contexts of application—technological or physical—or abstract, pure mathematics, i.e., devoid of any applicable context. The main learning goal in this context is to raise the implicit mathematical concepts and relations to conscious awareness by involving in a series of the UDGS phases through a bottom-up trail.

The UDGS model fits rather well in technology-based environments where students are both engaged in the construction of executable symbolic representations and in receiving informative feedback and naturally progress across the UDGS stages (Hoyles, 1986; Hoyles & Noss, 1987). In addition, Hoyles and Noss (1987) supported that the programming environments imply the need for formalization, due to its symbolic nature, which can foster the transition from using to discriminating or from using to generalizing.

Even though this model provided a strong theoretical tool for analyzing mathematical meaning-making, the cycle of research attention it accrued was rather short. It was before long replaced by different models, whose emphasis diverged from analyzing the shaping of mathematical meanings and, instead, gained a multidisciplinary or programming-oriented viewpoint for analysis (Benton et al., 2016). This could be ascribed to technological limitations of that era, which resulted in the temporal distancing of mathematics education away from programming (DeJarnette, 2019). As a consequence, the challenge of “transfer” from the computer-based situation to abstract mathematics remained unresolved, while the way that students move towards reflective phases of acting within a digital resource was not considerably empirically supported.

**Fig. 2:**
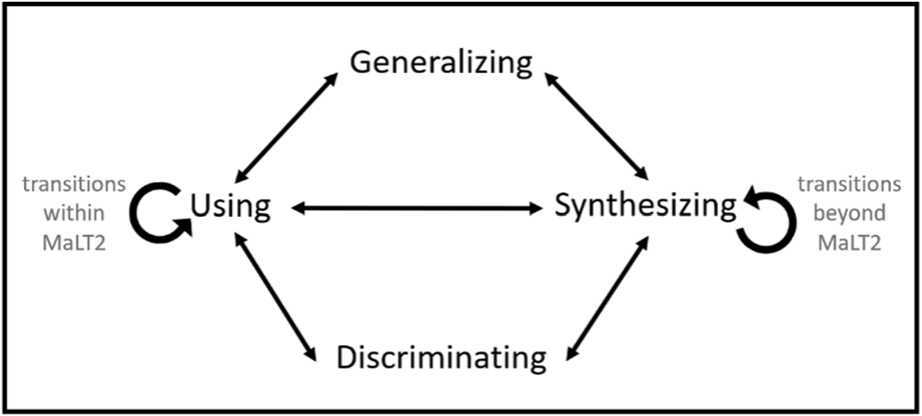
A modified version of the UDGS model

As computational thinking and coding have recently re-emerged as wide-ranging educational trends, we support that revisiting this long-standing challenge and reflecting on ideas around its theoretical foundations within contexts of new, emerging technologies and novel educational settings are more relevant than ever (DeJarnette, 2019; diSessa, 2018; Kynigos, 2015). Therefore, we consider important to look deep into the way mathematical meanings are formed and evolved within novel technological programming-based contexts that afford higher level of expressivity and multiple interconnected representations of mathematical concepts. The UDGS model seemed appropriate for framing our research objectives, since mathematical meanings are placed in the center of analysis. However, when starting analyzing the collected data, we found ourselves needing a modified version of it, one that would help us convey students’ dynamic process of mathematical meaning-making, as influenced and supported by the integration of different contexts of application and representation of mathematical ideas—within and outside the digital resource of MaLT2. Thus, we made some conceptual modifications for adjusting it to the results of our study and the digital environment used (Fig. 2).

First of all, we incorporated a type of “fluidity” inside the model, in the sense that students can navigate among each phase of the model multiple times. The UDGS model, as presented by Hoyles and Noss (1987), underlies a kind of linearity, with students starting with the using phase and gradually moving to the generalizing and synthesizing ones. We do not entirely oppose to this linear trail, since the overall meaning-making process entails a bottom-up flow towards generalizing. However, the proposed version enables the analysis of more possible routes, aiming at providing an articulate image on how students construct generalized meanings and capture their progression. Second, we place the using and synthesizing components in the center of the model and further conceptualize them in terms of transitioning among different representational contexts. Within this extended network of possible routes, we adjusted the conceptualization of each phase of the model within a broader perspective, as follows:

- The using component involves students using mathematical concepts as tools in either an intuitive or a reflective way, while transitioning among the different representations within the digital resource, in order to achieve particular goals. Students can therefore begin in this phase, but can also re-encounter it multiple times during their attempt to achieve their initial goal, even after discriminating and generalizing mathematical concepts. For this reason, arrows originating from all the other components point to using, representing the use of a mathematical concept at each different level of the meaning-making process, e.g., after a mathematical relation is discriminated or generalized. These arrows are double-sided, since the converse route can be also taken, representing, for example, generalizing a relation after intuitively using it within the microworld. We pay special attention to this phase, since it provides the “clearest window” for viewing students’ meanings under construction—it is when these concepts are in use through the digital tools that reveal how students think of them.
- The discriminating component is viewed as locally distinguishing and identifying a mathematical concept or relation initially interwoven within a specific part of the digital tools used. It thus involves consciously recognizing the mathematical concepts in use, responsible for the dynamic or visual parts of the artefact (figural or animated) in the microworld, without necessarily identifying the way/rule under which they affect it. It is tightly connected to the using phase, since it usually emerges after observing tools where mathematics is implicitly in use, as well as the synthesizing one, in case a connection to abstract mathematics leads to such recognition. It can also lead to generalizing the distinguished relation, without further using digital tools, or even emerge as a result of a generalized meaning.
- The synthesizing component in this version plays a wider, profound role for analyzing students’ process of meaning construction. It involves connecting the mathematical concepts used/represented in a specific digital resource with different contexts outside the digital representations. In our case, apart from abstract mathematics (i.e., abstract mathematical notions and relations), the artistic domains of dance and music were integrated as external contexts in the design of the task. We conceptualize the synthesizing phase as transitioning from one context to another. Unlike the UDGS model, students are anticipated to move to the synthesizing phase at any stage of their meaning-making process—even at the very beginning, when ideas for how to use digital tools are connected to an external context, e.g., the art of dance, and mathematical concepts behind them still being vague. In such cases, students can synthesize different aspects of mathematics, before having started using the digital tools and discriminating mathematical properties. Thus, synthesizing is linked to all the other phases of the model with double-sided arrows representing all possible routes towards—or originating from—the artistic contexts or abstract mathematics.
- The generalizing component is conceived in a similar way as in the original UDGS model, as extending and expressing mathematical relations and being able to recognize, use, and exploit them through the digital tools. Conceptually, it involves acknowledging generalized relationships among the mathematical concepts in use. It is connected to using and synthesizing via double-sided arrows, representing all possible routes. On one hand, generalizing is closely linked to abstract mathematics through synthesizing, as one can build on previously formed meanings of mathematical concepts for extending them in order to be applicable for using in a specific situation within a digital resource. On the other hand, just using mathematical relations—implicitly incorporating in a digital representation—could also lead to generalizing, through attempts to interpret unanticipated behavior of the tools. Generalizing is considered to be the most abstract phase of the model. Thus, it is seen as a precondition for one’s meaning-making process corresponding to a mathematical concept, relation, or property used within the digital tools to be considered well-rounded in order to be part of our analysis.

In the analysis, we center our focus on the using and synthesizing phases and further conceptualize them in terms of transitions within and transitions beyond the digital resource, respectively.

### Transitions Within and Beyond MaLT2

**Fig. 3:**
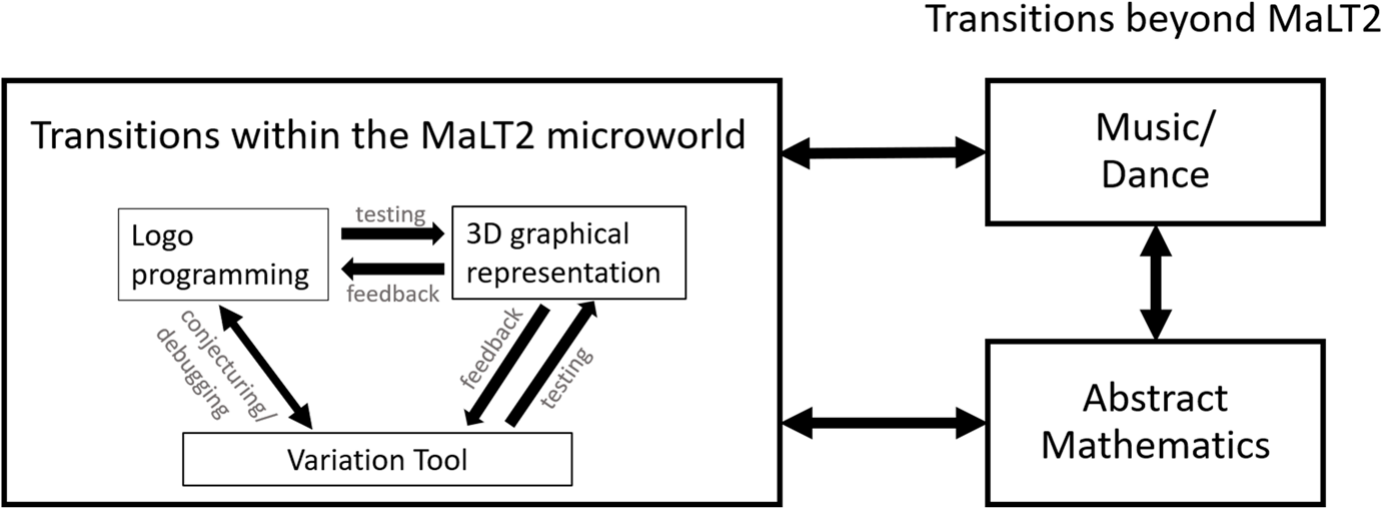
Transitions within and transitions beyond MaLT2

As already mentioned, this study is based on the assumption that transitioning among different representational contexts of a mathematized—at any level of abstraction—situation either within or external to the digital tools for achieving an artistic-oriented goal would enable a physical flowing among the UDGS phases towards generalizing mathematical meanings. Here, transition is conceived in two different ways: as moving between representations of the same mathematical object or property within a digital resource (transition within) or between the digital resource and the external contexts of art (music or dance) and abstract mathematics (transitions beyond) (Fig. 3).

MaLT2, the digital resource used in this study, integrates the representational contexts of variation tools for dynamic manipulation of parameter values together with Logo programming and Turtle Geometry. Thus, it provides a network of interconnected digital representations for modeling mathematical objects and relations that could support transitions within for the creation of a figural dynamic artefact. In addition, the supplemental contexts of music and dance, where mathematical ideas can get an esthetic and practical form, could also support the transitional process beyond MaLT2.

This conjecture also relies on a parallelly growing argumentation claiming that “music, when facilitated by multiple, intuitively accessible representations, can become a learning context in which basic mathematical ideas can be elicited and perceived as relevant and important” (Bamberger & diSessa, 2003, p. 123). This theoretical construct can help us view and conceptualize each student’s meaning-making process as a “flow of transitions” among the different contexts. We use this term to define a series of transitions within and beyond MaLT2 that enable students’ navigation throughout the UDGS phases. It can, thus, provide a tool for potentially discussing the way their mathematical meanings are progressively shaped and generalized. We further elaborate on each type of transitions in the next subsections.

#### Transitions Within the Microworld in MaLT2

**Fig. 4:**
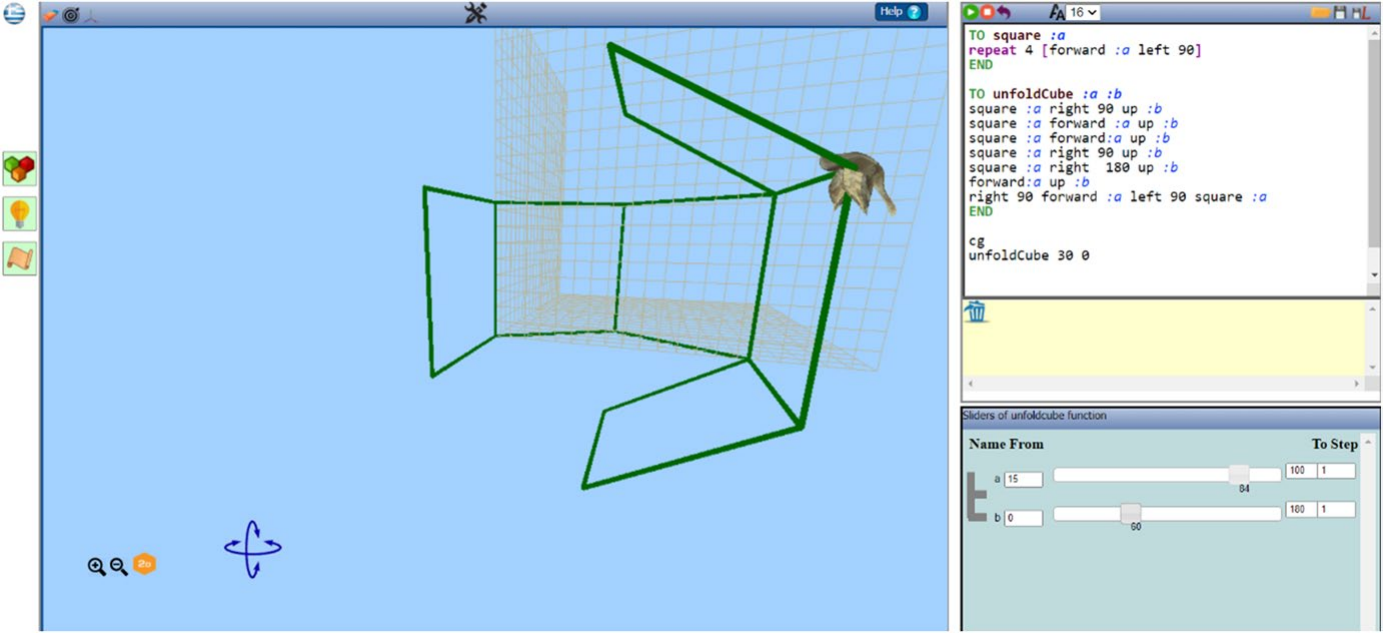
An example of an artefact within the three interconnected representational contexts of MaLT2 interface (http://etl.ppp.uoa.gr/malt2/)

Transitions of this type involve students’ moving among symbolic, visual, and dynamic virtual representations of mathematical objects or relations while using them to construct or tinker an artefact. They are represented through arrows in the left area of Fig. 3. They are held within the digital resource as a result of its three interconnected representational contexts:

- The Logo programming editor (up right area in Fig. 4)
- The variation tool (bottom right area in Fig. 4) for dynamic manipulation of each parameter value, corresponding to a parametric procedure, through dragging of its sliders
- The 3D scene (left middle area in Fig. 4), where two- or three-dimensional figures, as well as their dynamic transformation while using the variation tool, can be represented

The Logo programming editor can provide an open authoring system for users to code figural models. It supports Logo movement commands (see Table 1) and repetition/conditional commands, as well as parametric procedures and sub-/upper-procedure construction. By running movement commands, one can control the moving condition (orientation or displacement) of the avatar—whose default form is a sparrow. As the avatar moves, it leaves a colored trace which results in the construction of figures in the scene.

**Table 1:** Description and examples of the main Logo movement commands in MaLT2

Command	Description	Example
Forward number or fd number	Avatar moves forward as many steps as the number value	fd 80
Right number or rt number	Avatar turns its head to the right by as many degrees as the number value	Right 90
Left number or lt number	Avatar turns its head to the left by as many degrees as the number value	Left 75
Up number	Avatar turns its head upwards (looks up) by as many degrees as the number value	Up 120
Down number or dn number	Avatar turns its head downwards (looks down) by as many degrees as the number value	Down 30
Roll right number or rr number	Avatar rotates around itself clockwise by as many degrees as the number value	rr 90
Roll left number or rl number	Avatar rotates around itself anticlockwise by as many degrees as the number value	rl 180

**Fig. 5:**
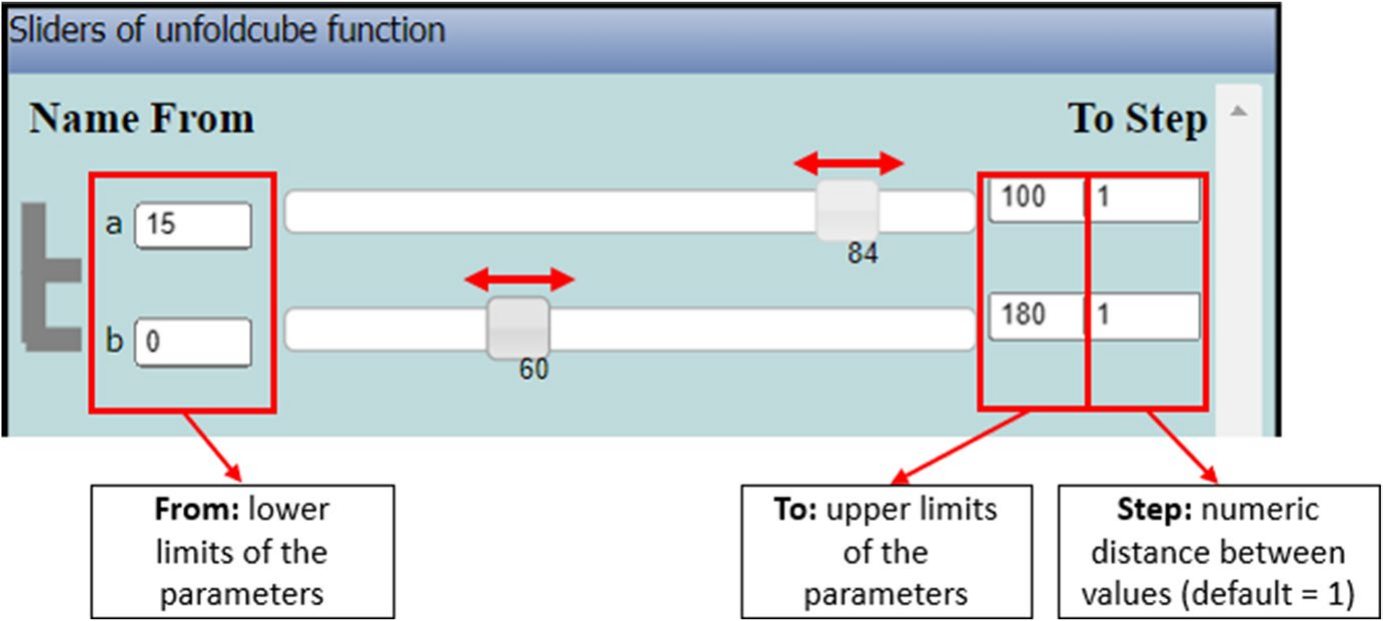
The variation tool with four fields of interaction: sliders, lower limit, upper limit, and step values

The variation tool appears when a parametric procedure is created in the editor. For example, in Fig. 4, the parametric procedure unfoldcube is written and run by the user. It has two parameters: *a* and *b* that in terms of the Euclidean geometry correspond to the length of each square’s side and the degrees of each turn between two consecutive squares, respectively. In the variation tool, two sliders have appeared: one for each parameter. By dragging one slider, the values of the corresponding parameter change and, simultaneously, the visual figural transformations of the avatar’s trace are shown in the 3D scene. Thus, a sense of dynamic “behavior” of a figural model is created. The user can interact with these dynamic figural transformations by either dragging the sliders (using the mouse or the right left arrows of the keyboard), or changing the lower and upper value limits of a parameter or the step value at which a parameter is incremented or decreased (Fig. 5).

While constructing or tinkering a figural model in MaLT2, transitioning among the three representational contexts physically emerges (Diamantidis et al., 2015, 2019; Grizioti & Kynigos, 2021; Kynigos & Diamantidis, 2021; Kynigos & Latsi, 2007). Both the editor and the variation tool provide interactive fields affording expression and experimentation, where students can use mathematical ideas as tools for constructing or tinkering an artefact. The scene, on the other hand, offers instant feedback providing reflection on these ideas. Transitioning within these three different representations of mathematical concepts in use is assumed to cultivate a continuous circle of formulating and testing conjectures, by using a mathematical idea in the interactive parts of the digital tools—in the editor or the variation tool—receiving feedback from the scene and debugging or extending the initial idea. This, consequentially, is assumed to lead to a physical fluidity among the UDGS phases, with the potential of provoking the generalization of these ideas.

#### Transitions Beyond MaLT2

This type of transitions corresponds to the synthesizing phase of the UDGS model and involves building of connections between the representational fields of MaLT2 and two external contexts: (a) the artistic context of music and dance, where mathematical concepts get an aesthetic and practical form and a physical (acoustic, visual, or dynamic) interpretation; (b) abstract mathematics, consisting of the abstract mathematical notions and relations, referring to abstract entities, which reside behind their context of application or representation.

Abstract mathematics does not necessarily correspond to the mathematical content and structure as it is inserted to the school curriculum, but could rather be an extension or a restructuration of it, or even connected to a different mathematical area outside the school curriculum. There are three types of synthesizing in this model, represented with double-sided arrows in the right area of Fig. 3 and are conceptualized as follows:

- Transitioning between MaLT2 and the artistic context; a student can transition from visioning an artistic idea, expressed in words, drawings, or physical gestures, to expressing it in MaLT2 by either interacting with the editor or the variation tool. Reversely, a student can transition from observing a figural model with subjectively intriguing behavior in the MaLT2 scene to forming or expanding an artistic idea represented in words, drawings, or gestures.
- Transitioning between MaLT2 and abstract mathematics; a student can either transition from forming a mathematical idea in words or notes to expressing it in MaLT2 by either interacting with the editor or the variation tool, or, reversely, transition from observing a figural model with subjectively intriguing behavior in MaLT2 to forming or extending a mathematical idea represented in a symbolic notation through words or notes.
- Transitioning between abstract mathematics and the artistic context. A student can transition from one representational context to the other, without using the technological tools, through paper-and-pencil investigation communicated through words, notes, drawings, and gestures.

Synthesizing is thus viewed as transitioning between two different contexts that represent a mathematical idea in a different way, notation, and level of consciousness and abstractness. In order for students’ meaning-making process to progress towards discriminating and generalizing phases, transitions beyond MaLT2 to the context of abstract mathematics are essential. This should not degrade the importance of transitioning to the artistic context, since it is assumed to be vital for grounding and guiding the whole process.

Music and dance, in particular, embed multidisciplinary notions such as periodicity, symmetry, harmony, and synchronicity, which possess both an artistic and a mathematical aspect and can be easily represented with computational tools. Thus, they can afford synthesizing through establishing links between their different aspects, either abstract or in use. We presume that this artistic context, as being closer to students’ intuition, sensibilities, and interests, would provide a source of motivation and creativity fostering the natural fluidity throughout the model guided by their own agency.

### Research Questions

Relying on the theoretical lenses described in the previous sections, we set out two main research aims: to shed light on (a) the way transitions among different contexts of the modelized network influence students’ meaning-making process in terms of the UDGS model and on (b) the nature of mathematical meanings that would emerge from this open artistic, digitally, and programming-based activity. Thus, we pose the following research questions:

- What role do transitions within and transitions beyond MaLT2 play in the progression of the students’ meaning-making process?
- What kind of mathematical meanings are derived out of the artistic contexts of music and dance through these transitions?

## Design of the Research

In order to answer the above questions, we engaged in design research through an implementation with one small group of students, which took place in a school classroom in Athens, Greece. The group consisted of three Grade 8 (aged 14–15) students (Mary, Nikos, and Chris)^1^ who volunteered in an after-school setting. A former pilot implementation of the study provided feedback for small changes in the current one, as part of the design-based research (DBR) framework that we adopted (Bakker, 2018). We consider these two small implementations as the beginning of a circle of design experiments in a bigger scale, where we aim at re-adjusting the theoretical and design ideas of this study.

The main activity designed for this study was titled “Dancing Animations.” We prepared a list of fourteen song extracts,^2^ each one of which being considerably cropped in order to have the same, steady musical rhythm throughout its duration. The aim of this activity was for students to construct an animated figural model of their own ideas in the MaLT2 microworld and synchronize it to the rhythm of a song of their option, in order to create a dancing animation. The animating feature can be carried out in MaLT2 through dragging of the sliders of the variation tool that creates a sense of dynamic “behavior” to a figural model constructed by a parametric procedure. Their final creation would be controlled by them through the sliders of the variation tool,^3^ while it would be captured with a screen recording application. After the completion of the research, the song audio sound would be added to the video in order to create the final product. No video mixes or trimmings could be made.

Due to Covid-19 restrictions, each student was working on a different lap-top, using headphones and keeping notes in a separate paper sheet. However, they were encouraged to discuss ideas or asking for advice within their group. All students had some previous experience with MaLT2, but for revising reasons, an introductory task was embedded into the activity in order for them to recall MaLT2 commands and functionalities. It was named as a MaLT2 “warming-up” and was structured by the following three questions:

1. Can you make a rectangle move in the plane?
2. Can you make ten rectangles move in the plane simultaneously?
3. Can you make ten rectangles move in the 3D space simultaneously

The results taken from this initial task will not be discussed in this paper, since its purpose was preparatory. After its completion, students were encouraged to engage in the main task of creating and synchronizing the dancing animation. The task had a profound level of freedom so as to stir up students’ own agency and provide authentic data corresponding to the openness of our research questions. The whole activity was planned to last for 3 h. The second author was the researcher who participated in this setting and took on the role of facilitating the progress of the activity, helping with functional problems, and provoking students to express their ideas out loud. The questions used for this reason were not pre-structured, but were rather adjusted to each student’s flow of transitions.

## Methodology

The data for this study consisted of students’ discourse, actions in the digital resource, MaLT2 saved files—including the final artefact of each student—and paper-and-pencil notes. The recording tools were a screen video and audio recording application for both the computer screen—showing the MaLT2 environment—and its audio outputs. The latter was used for capturing the sound of the song, which was played in an audio player application, controlled by each student’s initiative. The same recording application was used for capturing external sounds (mainly for students’ discourse). The output of this application, which was the main data unit for analysis, was a 3-h-long video. Another source of data was students’ gestures and facial expressions throughout the activity, which were monitored and noted down by the attendant researcher.

We analyzed the data adopting a qualitative approach, from which the modified version of the UDGS model was emerged. Students’ actions and expressions—either linguistic, gestural, noted down, or captured through their activity within the digital resource—were initially put into categories. Each category corresponded to one out of the five representational contexts (see Fig. 3), in order to capture their flow of transitions within and beyond MaLT2, forming three separate trails. Transitions within were traced directly through the video recording by following students’ actions with the digital tools, whereas transitions beyond were mostly traced through oral, noted down, or gestural expressions. Consequently, we identified instances of their flow as being mapped to phases of the UDGS model, in the way they were described in the “Theoretical Background” section.

As a result, each student’s overall captured activity was analyzed into phases of the UDGS model according to the way they were acting or communicating the intension of their action. For example, the using phase was more easily detected since it was directly connected to students’ decisions and actions mirrored through their transitions within MaLT2. It was initially assumed that every action in MaLT2, either intuitive or reflective, was linked to the—either subconscious or intentional—use of mathematical concepts, given the inherent highly mathematized way of using MaLT2 tools and functionalities. In the case of using mathematical concepts intuitively, the researchers interpreted students’ actions based on their subsequent more intentional actions and by employing their own insights and agency.

On the other hand, tracing the discriminating, synthesizing, and generalizing phases was connected to a deeper level of analysis focusing on students’ discourse, notes, and gestures accompanying their transitions within MaLT2. The synthesizing phase included students distancing themselves from the computer by observing, discussing, and reflecting on the outcome, or writing down algebraic or geometrical notation, or even by making gestures linked to artistic—mainly dancing—gestures, and vice versa; and students returning back to using the digital tools after reflection. The discriminating and generalizing phases were both mirrored in students’ discourse, mathematical notes, and their way of using the programming language in the MaLT2 editor.

Discriminating corresponded to clearly recognizing the mathematical concepts in use by concretely referring to them, while generalizing was matched to expressing acknowledgement of generalized relationships and their utility in the digital construction. In some cases, students’ intensions remained quite vague until they achieved or gave up their initial goal and communicated the reasons that led them to do so. As a result, coding and categorizing their actions was a long back-and-forth process, the full details of which would exceed the space limitation of this paper.

In the following section, we present instances of four different flows of transitions generated from the students. The reason these specific flows of transitions were chosen was that they included all four UDGS, forming a well-rounded corresponded to different mathematical concepts. Each flow was labeled after a specific artistic idea which was set out as a guiding goal from the student and incorporated different kinds of mathematical ideas.

## Results

### Setting Up—MaLT2 “Warming Up” Task

The data analyzed for this study begun from the point where each student had completed the initial “warming up” task, which lasted 30 min, having constructed two procedures: (a) one that creates a rectangle with two parameters used for its sides; (b) one that creates ten rectangles by including the previous procedure. These rectangles were moving in 3D space while dragging the sliders of the variation tools. This was achieved through the use of one or more parameters in turn commands, i.e., “right” or “left” and “up,” “down,” “roll_right,” or “roll_left.”

**Fig. 6:**
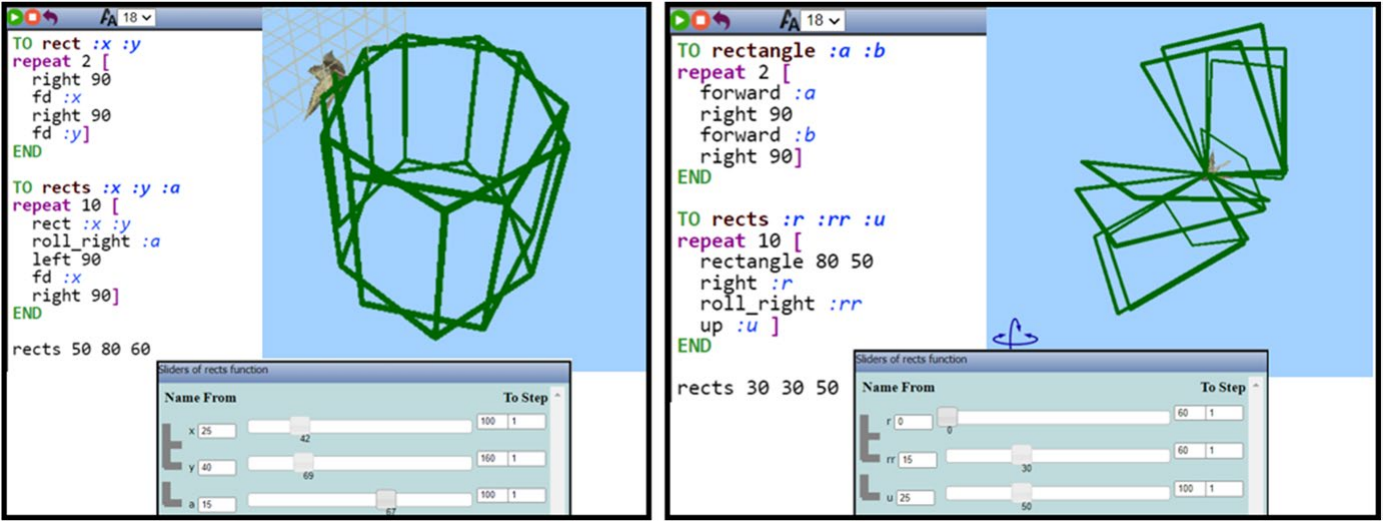
Instances of two artefacts made by two students (Nikos and Mary) constructed at the initial “warming up” task: the code was translated in English, since these students were writing programming commands in Greek

Each student used similar commands for the first procedure and quite different commands for the second one, which led to four individual animations. Examples of Nikos’s and Mary’s artefacts are shown in Fig. 6. A main realization drawn out of this task and shared by all three students was that, in order for an artefact to be animated, the use of the variation tool and, consequently, the use of parametric procedures are necessary. The constructed artefacts were the starting points for each student to begin with for making their figural dancing animation. They were free to make any changes to their already made artefact, either slightly ones or completely changing it and starting their creation from scratch. During the main activity, they had their picked-up song playing and pausing in the background and putting on and taking off their headphones occasionally.

#### Flow of Transitions for Making a Periodic Spinning

All four students followed a similar initial pattern of transitions within and beyond MaLT2, starting from dragging the sliders of the variation tool and observing the visual outcome at the screen to picturing a dancing move while listening to their song. Thus, all flows of transitions begun with intuitively using implicit mathematical ideas underpinned in the use of the variation tool, which was directly connected to the synthesizing phase, by connecting the visual outcome to an artistic idea. After dragging the sliders of the variation tool (using the keyboard left and right arrows) with the initial (default) input values, they tried different lower and upper limit values to each parameter while starring at the animated artefact. Mary’s flow of transitions was founded on an initial synthesizing phase, where she set the artistic goal of making dancing move resembling to a periodic spinning. She shaped and gradually generalized meanings around periodicity of angles.

**Fig. 7:**
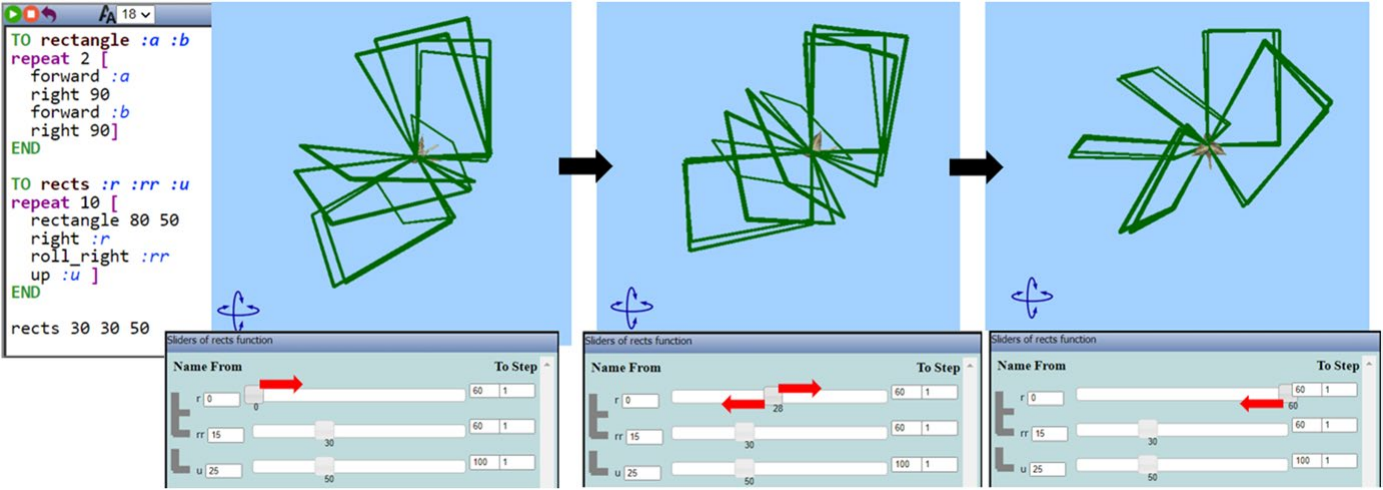
Instances of Mary’s initial actions using the variation tool by dragging the slider of the parameter *r* to the right and to the left alternately

Mary’s way of dragging seemed spontaneous at first, but gradually turned into a periodic dragging to the right and to the left, from the lower to the upper limit and vice versa (Fig. 7). She was simultaneously listening to the selected song extract (“Milky Chance—Stolen Dance”). A question posed by the researcher provoked her to communicate her thoughts:

Researcher: How could you make a nice dancing move? What do you think of that?

Mary: I like the way it spins when I drag the bar of the r. It is kind of, like dancing! To the right, and to the left. Nanana, nanananananana, nanananananana. It suits the song!

Mary transitioned beyond MaLT2 by discussing the “style” of her animation being suited to the selected song, while dragging the slider of the parameter *r*. She intuitively used the notion of periodicity integrated in the “right:r” command and the use of the corresponding slider, by repeating dragging it to the right and to the left periodically while repeating the words “right” and “left.” As her actions and words indicate, she discriminated that this parameter stands for the value of degrees that the avatar turns to the right, after constructing each rectangle, creating a pleasing dynamic outcome. She changed its upper limit from 60 to 100. She repeated dragging the slider from the left (*r* = 0) to the right until the upper limit (*r* = 100). She then changed it again to 120 and repeated the procedure.

Researcher: Why did you change it?

Mary: Because the higher the number, the more it turns around.

Researcher: What turns around?

Mary: The rectangles. Turn around each other. (...) Actually they turn around the bird.

Researcher: And what kind of dancing move are you thinking of?

Mary: It looks like spinning. I want to make it spin more. (...) To look even better.

**Fig. 8:**
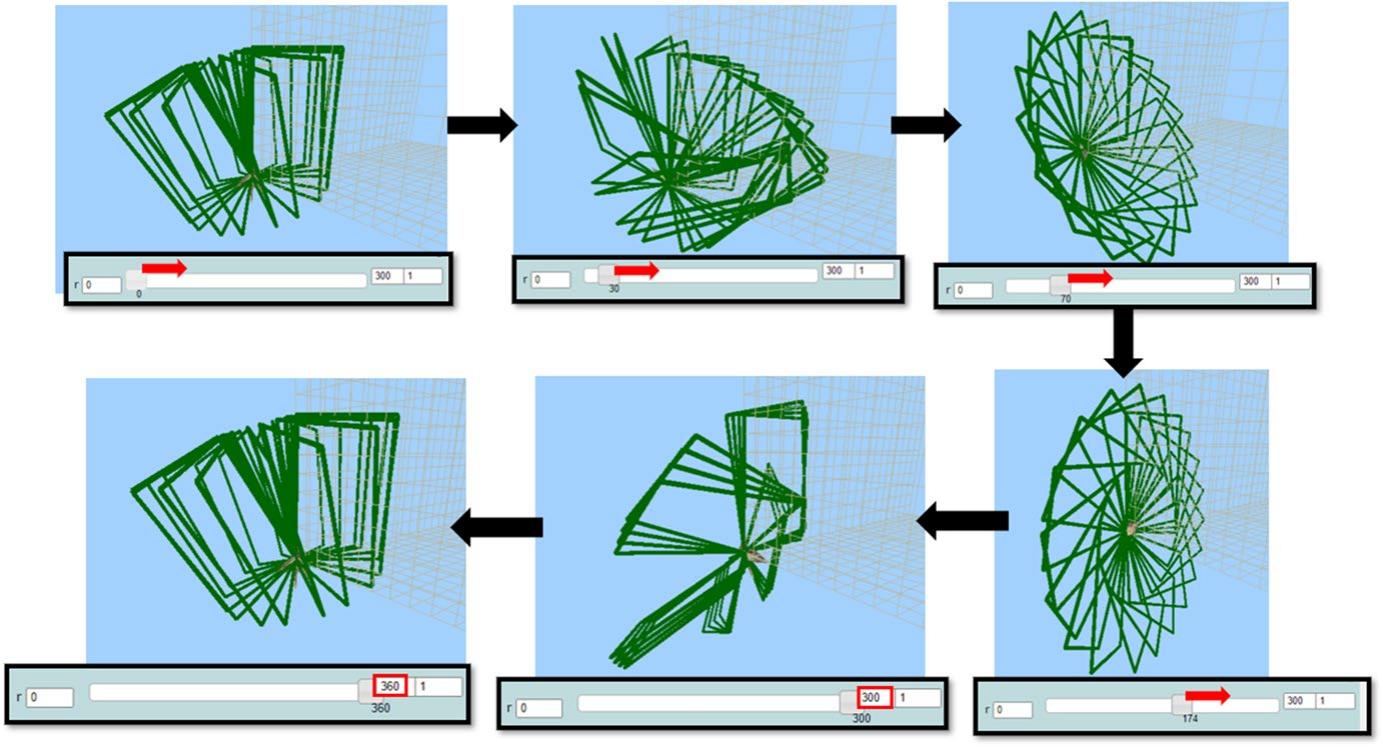
Instances of Mary’s animation while dragging the slider of the parameter *r* to the right (when rr = 37 and *u* = 10)

Mary intuitively approached the intrinsic periodicity of the parameter *r* of the command “right:r.” After transitioning beyond MaLT2, towards the artistic context, she set out the goal of creating a spinning dancing move. She proceeded by seeking for a way to achieve it through transitioning within MaLT2. As it was shown by her subsequent actions, she conjectured that by changing the upper value of the parameter *r*, she could increase the spinning duration. She started gradually increasing the number at the upper value input box from 100 to 180, from 180 to 200, from 200 to 300, and then from 300 to 360 (Fig. 8).

Every time she made a change, she was testing the result through transitions within, from using the variation tool to getting feedback from the dynamic outcome at the scene and vice versa. After a while, she noted down “1 circle = 360.” The following dialog reveals how her transitions within MaLT2, where she intuitively used the concepts of angle and circle, led her to transitioning beyond MaLT2, to the abstract mathematics context where already formed mathematical meanings were recalled. She progressively discriminated the mathematical properties of the “right” command connected to the concepts of angle and circle.

Researcher: How did you find this value? (showing the value of 720 as upper limit). Can you describe your way of thinking?

Mary: I tried different values. I put right 180 because I thought 180 degrees is the bigger turn. While it goes from 0 to 180 it was like starting spinning from the left and then slowly turning to the right while opening like a flower. But then I tried 200 and saw that it turns even more. This is not actually an angle, it is a turn. It is different. (...) When I put 300 it starts spinning again to the left while closing. And when it becomes 360 it ends where it begun!

Researcher: How did you find the exact value 360?

Mary: I put 300 and show that it almost got its initial form. So, I thought of a circle, a full circle, which is 360 degrees. (...) 360 just makes sense. It is a whole turn, starting from this shape, spin all around and return to this state. Now it’s like it did a whole circle of spinning.

**Fig. 9:**
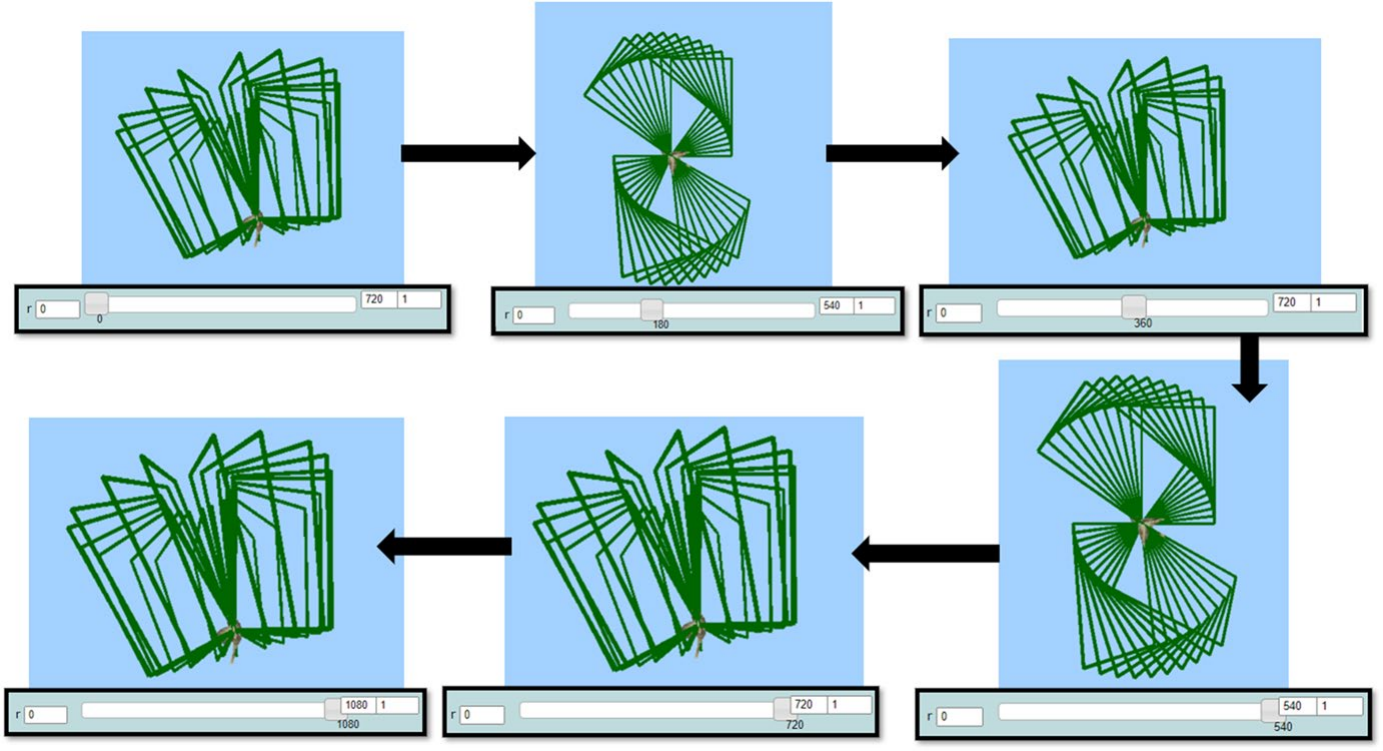
Instances of Mary’s animation while changing the upper limit and dragging the slider of the parameter *r* to the right (when rr = 37 and *u* = 10)

After using and experimenting with different values, she ended up synthesizing digital and mathematical aspects of the artefact’s behavior, by making the connection between the dynamic manipulation of the parameter *r* and the mathematical concepts of angle and circle. This transition beyond came up naturally after continuous transitions within MaLT2, through a circle of conjecturing and testing on the variation tool and getting feedback from the dynamic outcome in the scene. She also intrinsically discriminated the periodic property of angles and consciously used it to create a periodic motion. Mary further expanded and generalized mathematical meanings around periodicity of angles by posing the following question, which set another round of transitions within and beyond (Fig. 9).

Mary: What would happen if I put more than 360 here?

She tried the value of 400 in the upper limit of the parameter *r* in the variation tool. She dragged its slider and took a confirmatory expression. Then, she noted down the calculation: “360 + 180 = 540.”

Researcher: Why did you make this calculation?

Mary: Because I want to try what will happen if it goes through another circle. It restarts spinning in the same way after 360, see? (...) I bet that at 540 it will be open again. At the open position. (...) Yes, I knew it! So now if I try ... 720 it will close again like in the initial position. Yes! Every time I will add another 360 degrees, it will go another full round.

Then, she used the value of 1080 and confirmed her conjecture:

Mary: OK, know I get it. It is because 360 is a full circle.

Researcher: How can you use that in your animation?

Mary: (...) I can put as many full circles I want because the figure does a new full circle at 360, 720, 1080. That is all the multiples of 360. (...) Then it will be like dancing, spinning and spinning all over again.

Mary after reflecting on the feedback received through MaLT2 3D graphical representation, transitioned beyond MaLT2, to the context of abstract mathematics, in order to mathematically interpret the behavior of her artefact. She was led to the generalization that the period of the “turn right” function, connected to the concept of angle, is 360°. She also synthesized this generalization with its artistic aspect by transitioning beyond MaLT2, to the artistic context, and appreciated the physical dynamic outcome. She extended her generalized meanings around mathematical properties emerging from the periodicity of angles, as the following sharing of her thoughts indicates:

Mary: It is like from 0 to 180 is goes one way, then from 180 to 360 it goes the reverse way.

Researcher: What do you mean it goes the reverse way?

**Fig. 10:**
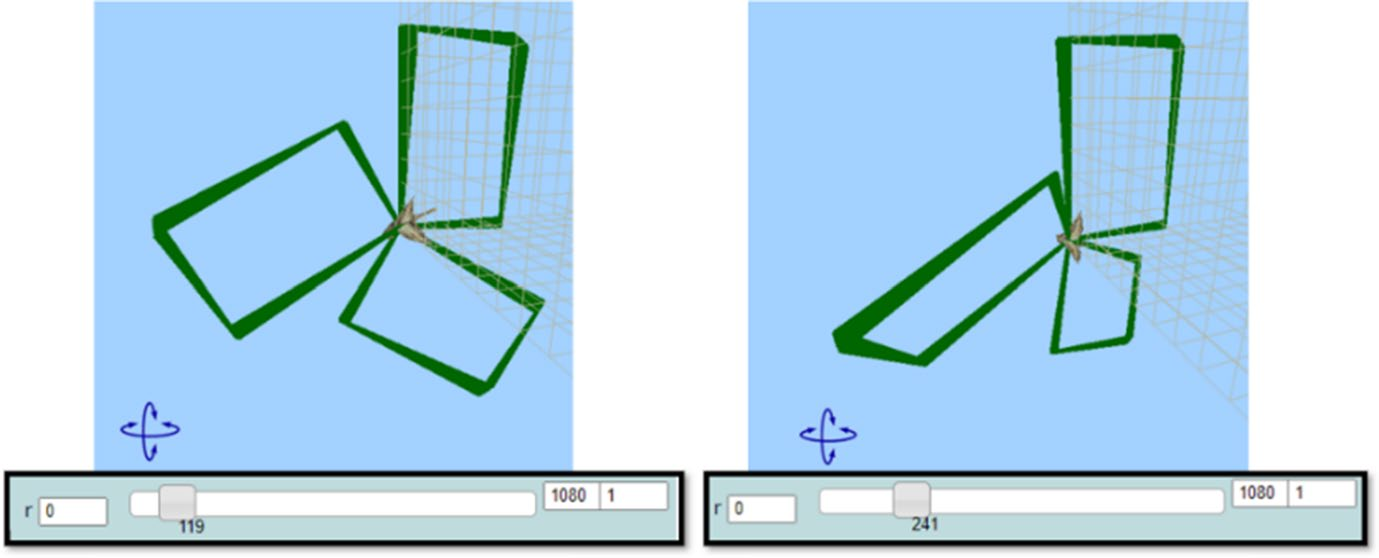
Instances of Mary’s animation when *r* = 119 and when *r* = 241

Mary: I mean the same way it spins while it opens, the same way it spins while it closes. I don’t know how to explain it. Now, look, when r equals 119, it looks like this, like a windmill. It looks exactly the same but at the opposite side. (Fig. 10)

Researcher: What is the value? Do you think there is a relationship between them?

Mary: The value is 241. 119 and 241. Maybe there is something to do with 360? Ah, yes! Because 241 plus 119 equals 360. (...) So every time two values together equal 360, the figure is the same but reversely.

She then used two more pairs of values whose sum is 360 and verified her conjecture (Fig. 10). Mary further synthesized the mathematical, digital, and artistic aspects of the generalized meanings around periodicity. The following part of a dialog reveals her respective transitions beyond:

Mary: The dancing move now has three circles of spinning like this and reversely...if I start dragging from 0 to 1080. (...) I have to find how many circles are there in the song.

Researcher: What do you mean by circles?

Mary: I mean how many repeats are there in the song. Because both the song and the dance should have the same repeats. (She listened to the song extract three times.) Each circle ends with the lyric “we don’t talk about it”. The song has four whole circles. So I extended the value here by 360. 1080 plus 360 is 1440. Now the dancing circles are also four.

At this point, Mary synthesized her meanings around periodicity by transitioning among its mathematical, digital, and artistic representations. She established connections between the period of the song, the period of the dancing move, and the period of angles. She consciously expressed that the concrete number of periods of the song and the animation should be jointly taken into account in order for them to match together. She finally used the synthesized meanings in order to synchronize her dancing animation to the musical rhythm.

#### Flow of Transitions for Tuning the Periodic Spinning into the Musical Rhythm

Building on the previous flow of transitions within and beyond MaLT2, Mary set out a new goal based on the initially intrinsic idea of synchronicity. She listened to the song extract, while dragging the slider of the parameter *r* to the right. The following dialog indicates her way of thinking:

Researcher: How can you say if your animation is in the musical rhythm?

Mary: It is not. I can say it. Because it is too slow. I want it a bit faster, but I don’t know how.

Researcher: Why faster? What do you want to achieve by that?

Mary: I want ... every time the song says “we don’t talk about it”, I want the dancing move to be completed and restarting. Because that is when the song circle ends. I want to make them repeat together. (...) So that the four circles will match together.

Mary transitioned from using the variation tool and observing the animated outcome (transitions within) to the artistic context (transition beyond) for setting out the goal to tune her animation into the musical rhythm, in terms of speed. She posed the problem that the animation is “too slow” for being in tune with the song and explicitly expressed the need to match the “music circle” to the animation one. Without using the formal terms, she implied that she wanted to adjust the periods of the song and the dancing animation by reducing the latter. Thus, an implicit transition beyond MaLT2, between the artistic and the mathematical context, was made. She continued by consistently listening to the song and dragging the slider of the parameter *r*, transitioning from the digital dynamic representation to the musical rhythm (transition beyond).

Researcher: How much faster do you need it to be?

Mary: I don’t know. Not much faster. The song circle finishes when r equals 250.

Researcher: So, you want the animation circle to finish at 250 too, right? What do you think you should change? Something at the variation tool or the procedure’s code? Where?

Mary: The code. I will change the command ‘right r’. (...) It has to make a full circle when r becomes 250.

Mary went through a circle of transitions within MaLT2, where she intuitively used mathematical notions around synchronicity and rhythm. She went from forming a conjecture by using mathematical notions of additive relationship and proportion; to experimenting this conjecture within MaLT2 representational fields; and finally transitioned from MaLT2 to the artistic context (transition beyond) for testing its validity according to its level of tuning. The way of using mathematical notions within the digital tools gradually turned into a conscious intentional one, leading to discriminating the mathematical ideas.

**Fig. 11:**
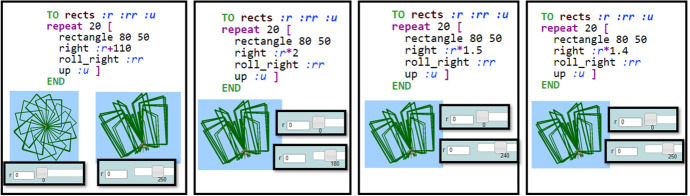
Instances of Mary’s transitions within MaLT2

As shown in Fig. 11, she changed the command “right:r” to “right:r + 110,” then to “right:r + 300,” then to “right:r*2,” then to “right:r*1.5,” and lastly to “right:r*1.4.” When the researcher returned for discussion, she had noted down the calculations “360–250 = 110” and “360/250 = 1.44.”

Researcher: Did you make it?

Mary: Yes, this time I’m sure!

Researcher: Can you explain your thinking? (...) How did you end up writing r times 1.4? Why did you do these calculations?

Mary: At first, I wrote :r + 110 because I thought that the circle would end at 250. Because 250 plus 110 equals 360. But then I saw that when the circle starts (...) when r equals 0 it is not at its initial form. Closed like this. It was not a full circle. So, then I thought that no matter what I would add to r, it doesn’t get any faster, it just starts at another state. (...) Then I tried to multiply it with 2 and saw that it does start at its initial form and that it was a lot faster! But it was too fast. Its circle was ending at 180, not 250. So, I tried different values and I found 1.4 is the right one.

Researcher: How did you find it so accurately? Did you try so many numbers?

Mary: I tried some numbers and I was getting closed and then I calculated that 360 divided by 250 equals 1.44. And it worked!

Researcher: Do you have any idea why it worked?

Mary: Because when r becomes 250, it turns right 250 times 1.44; which is 360. So, a full circle! But now each circle is shortened. (...) This is why it is faster now

The above dialog reveals the way Mary’s transitions beyond MaLT2, where she synthesized meanings among digital, mathematical, and artistic representations of a period, led her to discriminating and generalizing phases. While transitioning within the representational contexts of MaLT2, she discriminated the functional relationship between the input values of the slider and the right turn as means of adjusting the animation’s speed. She used the additive relationship, transitioning from mathematics to expressing and testing it within MaLT2. After disproving her conjecture, she alternatively used the proportional relationship, testing different values of the multiplying parameter in order to adjust the period of the animated artefact. Transitioning within MaLT2 was catalytic for her to find the appropriate value. As she stated later, she found the value of 1.4 empirically and afterwards transitioned beyond MaLT2, to the abstract mathematical context, to making the division 360 over 250 and typically support her empirical choice. Thus, she finished this flow of transitions by expressing a generalization of this value being mathematically verified and synthesized it to the notion of the “shortened” period.

#### Flow of Transitions for Making a Periodic “Tango Move”

**Fig. 12:**
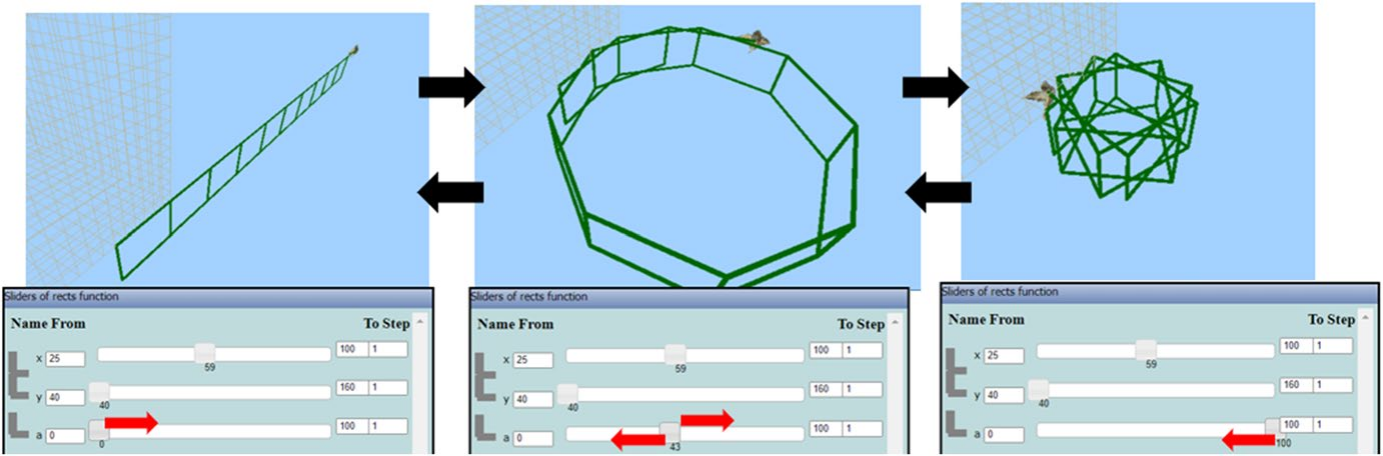
Instances of Nikos’s actions using the variation tool by dragging the slider of the parameter *a* to the right and to the left alternately

Nikos’ flow of transitions started with dragging the sliders of the parameter *a* of the procedure “rects” (Fig. 5) to the right and to the left repetitively, incorporating a periodic trait (Fig. 12). After continuously transitioning from the variation tool to observing the artefact for a while (transitions within and beyond), Nikos started visioning a dancing move more concretely. As shown in Fig. 12, when the parameter *a* equals zero (*a* = 0), the rectangles are aligned in the same plane, while as the values of the parameter *a* are reaching the upper limit (*a* = 100), the rectangles create a “wrapping” sense. These instances of the animated artefact were a bit surprising for him, as he seemed unsuspectingly excited for his creation. The following observation revealed Nikos transitioning beyond MaLT2 to describe his image of the dancing animation, which followed the transitions shown in Fig. 12.

Nikos: It’s like a tango move! Like when the couple opens their hands and then the man twists and closes the girl in his hands.

While expressing this artistic idea in words, Nikos tried to imitate the tango move with his body. He extended his arms horizontally and then wrapped his right arm towards the other. The transition from the variation tool to the artistic context (transition beyond) in order to form a new artistic idea of a dancing move set out the beginning of his experimentation. He then projected his artistic idea to MaLT2 graphical representation (transition beyond), by visioning it as a rhythmically “wrapping” and “unwrapping” of the artefact. He extended his goal for improving the animation, while listening to the song extract he had selected (“The Animals: House of the Rising Sun”) and simultaneously dragging the slider of parameter *a*. He shared the new goal that he sent out:

Nikos: When I play the song along with the animation, it seems too short. I need to make the animation last longer. (...) a is a turn. I can make it turn even more by increasing this. I can put 200 instead of 100.

Nikos synthesized the musical and digital contexts of his synchronization goal and used the notion of the period in order to connect the musical and animated rhythms. He transitioned beyond MaLT2, between the artistic-musical context and the digital dynamic representation. He expressed the goal of extending the animation’s period through posing the problem of their mismatched duration.

**Fig. 13:**
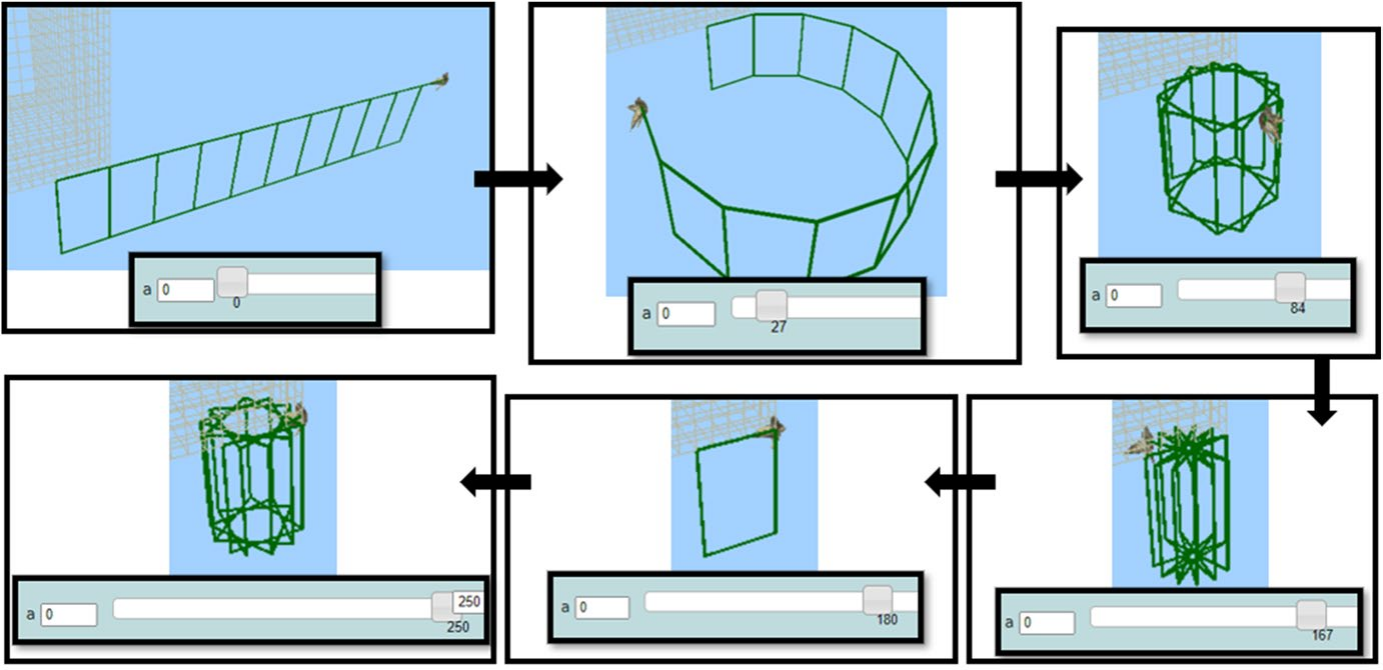
Instances of Nikos’s animation while dragging the slider of parameter *a* from 0 to 250

Then, he engaged in transitions within MaLT2, between the variation tool and the 3D scene, where the concept of period was used and investigated, from an intuitive to a gradually more conscious way. He changed the upper limit of the parameter *a* from 100 to 200 and observed the dynamic outcome while dragging its slider from 0 to 200 and back. Then, he changed the upper limit to 250 and repeated the process (Fig. 13). He made the following observation:

Nikos: After the value 180, it starts unwrapping again, but in the opposite direction. 181 is when all the rectangles are one on top of the other. This matches the tango move, I like it.

Nikos transitioned many times from the digital to the artistic context, by implicitly using mathematical ideas in his interaction with the tool, while trying to visioning his dancing move. He kept changing the upper limit of the variation tool to extend the animation’s period and observing the graphical outcome (transitions within), but he always changed it back to 180, because of its artistic symmetry (transitions beyond). At some point, he discriminated the notion of the angle and gave mathematical sense to the dynamic behavior of his animation:

Nikos: We know the angle of 180 degrees. Yes, 180 is the accurate number. It is a full angle. (...) From 0 to 180 it makes half a circle of dancing.

Then, he listened to the song while dragging the slider and disappointingly shared that the animation was not synchronized to it:

Nikos: It is out of rhythm. The song is slower. (...) I want it to be fully wrapped right before he starts singing. At the 00.11 minutes. It is almost twice as fast, because it stops at the sixth second. (...) But I can’t put a higher value here (shows the upper value) because it starts... when it’s higher than 180 it starts unwrapping.

**Fig. 14:**
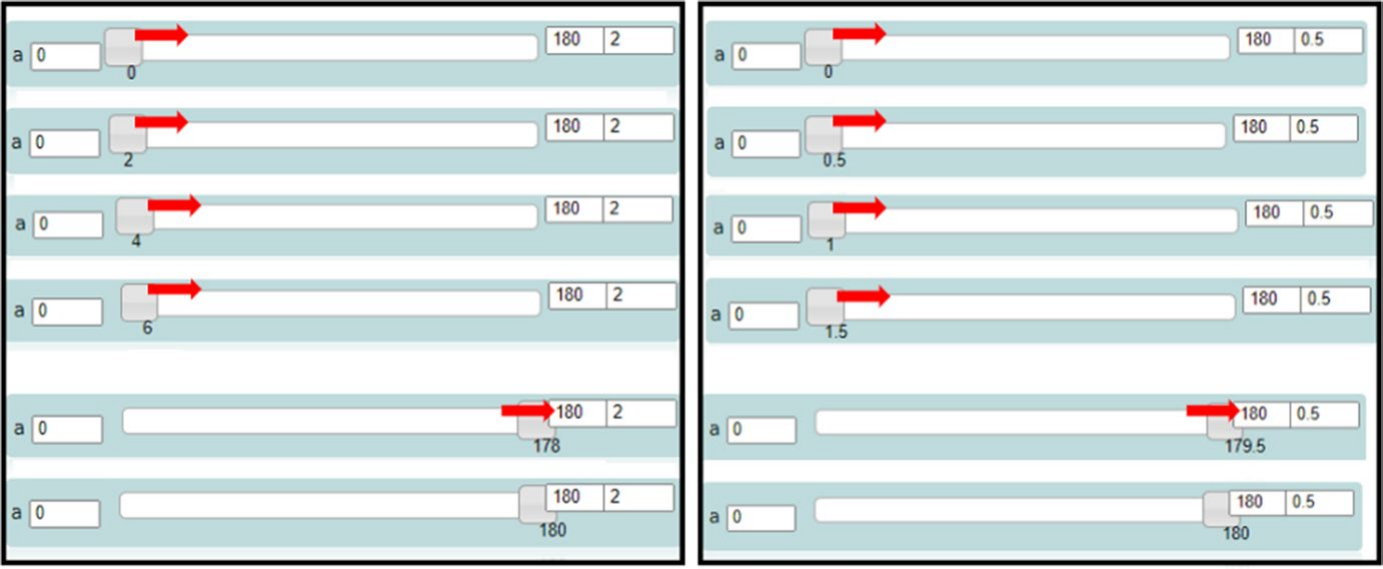
Instances of the variation tool after Nikos changed the step value to 2 (left) and to 0.5 (right), while dragging the slider of the parameter *a* (lowest and highest input values)

Nikos considered the first period of the song in terms of time and compared it with the period of the animation, synthesizing the notion period in the two different contexts and started discriminating their proportional relation. Then, he used the notion of the period and went through synthesizing phases by setting out the goal to adjust the animation’s period to the song’s one. After a while, he changed the step input value of the parameter *a* at the variation tool from 1 (default) to 2. He repeated the dragging of its slider from 0 to 180 (Fig. 14, left) and backwards while listening to the song. He was transitioning from the digital to the artistic context alternatively (transition beyond) in an attempt to discriminate how the change of the step value of the variation tool would affect the length of the animation’s period.

He shared such instances of discrimination, after testing whether the new animated artefact is synchronized with the musical rhythm through transitions within and beyond MaLT2:

Nikos: Now the animation is even faster. (...) If this is equal to two [referring to the step value] it means that a changes by two values. So, each time I press the computer key [right arrow], it skips one number. (...) The variable^4^ a takes half values, instead of 180. It takes 90 values. So, it’s four times faster than the song! It lasts only three seconds (...) Can I put a decimal number here?

Nikos then changed the step value of the parameter *a* from 2 to 0.5. Once again, he repeated the dragging of its slider from 0 to 180 (Fig. 14, right) and backwards while listening to the song. This time, he delightedly commented on the result:

Nikos: Yes, great! It is totally matched now!

Researcher:How did you manage to do it?

Nikos: I changed the step value to 0.5 now. I thought that it would make the animation last longer. The more the step value decreases, the more it lasts for the dancing move to be completed. Before it was taking 180 values, but now it takes twice as much (...) it takes 360 values. Thus, it lasts twice as much time, which is almost 11 seconds. (...) So now if you listen to the song, it matches perfectly. It starts wrapping slowly during the first song’s verse. And at the second one it slowly unwraps in the same rhythm. In the same direction as before.

Nikos’ meaning-making process started from intuitively using, then gradually discriminating, and finally generalized to some level the inverse proportional relationship between the step value and the number of input values of the parameter *a*, in order to extend the period of his dancing animation. He ended up establishing and using the exact arithmetic relationship between the step value, the number of input values, and the time duration.

#### Flow of Transitions for Making a 3D Whirling

**Fig. 15:**
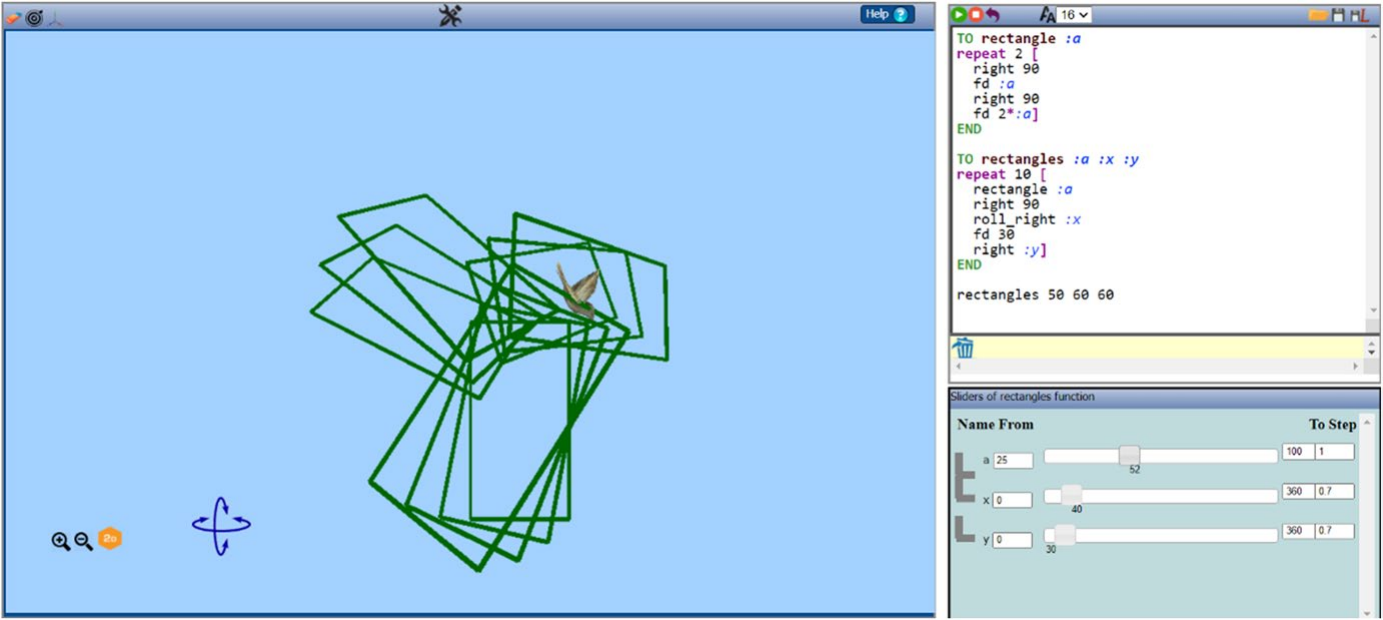
Starting point of Chris’s flow of transitions for making a 3D whirling dancing animation

Chris followed a sequence of creative actions close to Nikos’s one, where he ended up synchronizing his animation to the musical rhythm of the song he had selected (“Muse: Time Is Running Out”—30 s). Same as Nikos, he changed the step value of two parameters standing as input for “right” and “roll_right” commands many times until he found the most appropriate one (0.7) in order to adjust the animation’s period to the song’s one. During this flow of transitions, which will not be further analyzed here, he engaged in all phases of synthesizing, using, discriminating, and generalizing of mathematical meanings around periodicity, proportional, and inverse proportional relationship. Chris also engaged in another flow of transitions where he shaped mathematical meanings on the notions of the variable and covariation of quantities. The starting point of this process was a concern shared while dragging the sliders of the procedure “rectangles” (Fig. 15).

Chris: The problem is that when we will record this, I can only drag one slider. I won’t have time to drag both.

Researcher: Why is this a problem?

Chris: Because I like both ways of moving. When I drag the slider of y, it is nice because it is turning like a wheel, like a clock. And I like that because the song says “time is running out” and it is like a clock spinning. But I also like dragging the variable x because it is more...interesting. I want this variable to change, too. (...) Can I change them both at the same time?

Chris set out the goal to integrate the two different dancing moves in his animation. He transitioned from the digital dynamic representation to the artistic context (transition beyond), evaluating it with regard to the song’s style. He then transitioned from interacting with the variation tool to the abstract mathematical context (transition beyond) for giving mathematical sense to his goal.

Researcher: Can you think of a way? A way to change two different commands, these turns, simultaneously by dragging only one slider?

Chris: (...) What if I put the same variable? The variable y to both of them? Then I will only have one slider to drag.

**Fig. 16:**
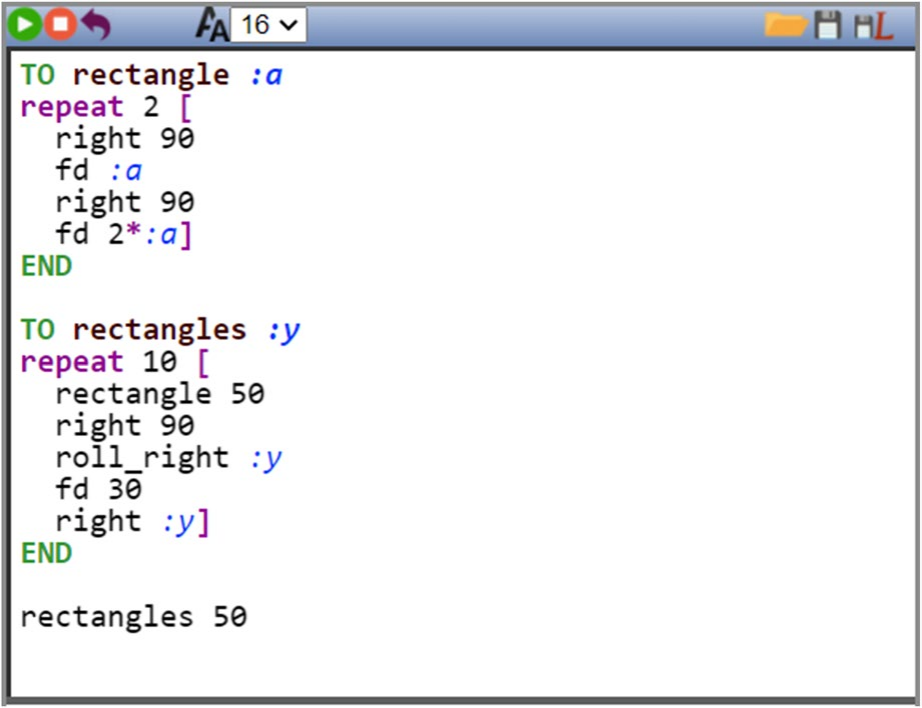
The new code after Chris made changes in the procedure “rectangles”

Chris had already used one parameter for four different commands at the warming up task, where he wrote the procedure “rectangle” using only one parameter (a) in all “forward” commands, embedding the proportional relationship of one side being two times bigger than the other. He proceeded by making changes in the procedure “rectangles.” He erased the parameters *a* and *x* and changed the commands “rectangle:a” and “roll_right:x” to “rectangle 50” and “roll_right:y” (Fig. 16).

He recognized that the command “rectangle” in the procedure “rectangles” can take a constant number rather than a parameter, since he was not interested in changing it. He started discriminating the role of the parameter, transitioning from the programming context of application to the mathematical one (transition beyond). After changing the code, he tested the dynamic outcome by dragging the slider of the parameter *y* and observing the 3D screen. He expressed his thoughts out loud:

Chris: Now it does both kinds of spinning simultaneously! It is much better! Look!

Researcher: Wow! Why do you think this is happening?

Chris: Because the same time that the angle of rolling right changes, the angle of turning right is also changing. I can control them both at once. So, it turns like this and like this together. (He turned his hand in two different directions.)

As emerging from his thoughts, Chris generalized his meaning on covariation of functions-commands with the same input. However, he did not stop his construction at that point:

Chris: I could make it turn right faster than it rolls right. I wonder how it will look like.

Researcher: What are you doing now?

Chris: I want to test how it looks if I make it turn right twice as much as it rolls right.

**Fig. 17:**
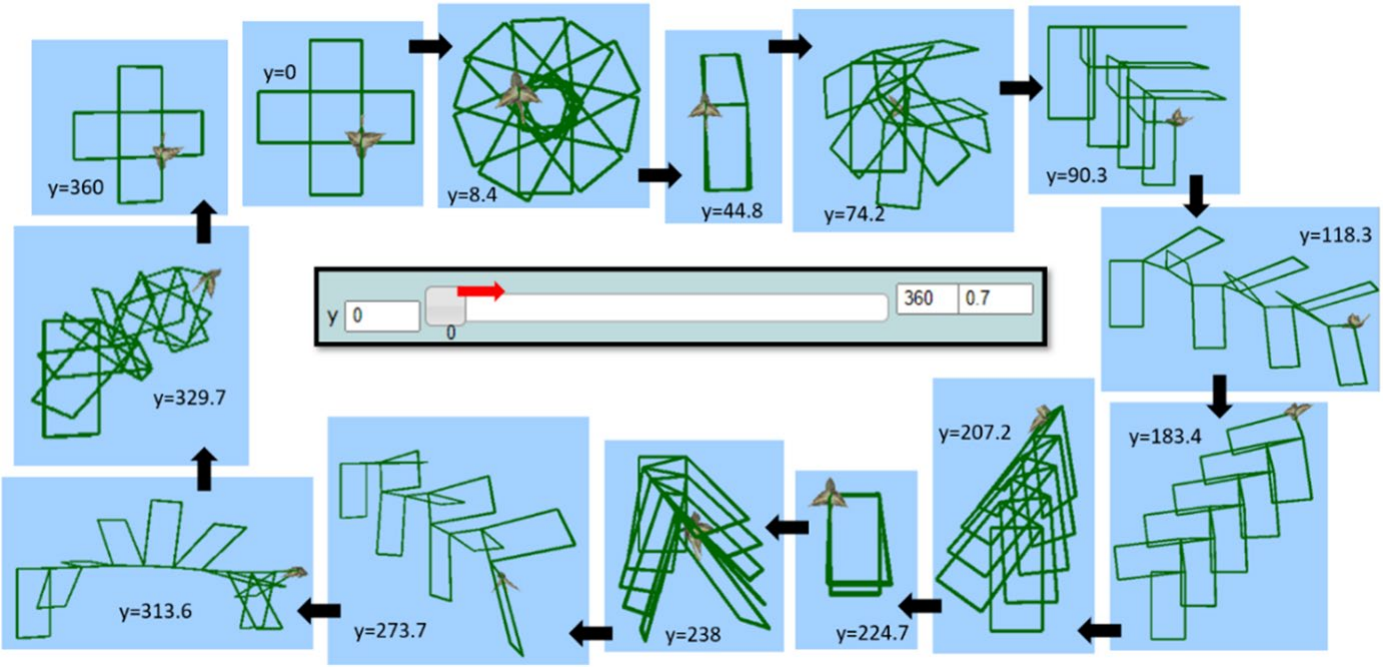
Instances of Chris’s animation while dragging the slider of the parameter *y*

Chris extended his artistic idea, set a new goal expressed in mathematical terms of proportional relation (transition beyond), and continued engaging in transitions within MaLT2, among all three representational contexts. He changed the command “right:y” to “right 2*:y.” He observed the dynamic outcome while dragging the slider of the parameter *y* from its lower to its higher limit and, reversely, while listening to the song (Fig. 17). He thus transitioned from the digital context to the artistic one (transition beyond) and was esthetically pleased with the result in combination with the song’s rhythm and style:

Researcher: Do you see any difference?

Chris: Yes, I like it better now! It moves more smoothly than before. It is like a weird 3D whirling... It matches the song a lot!

Chris: If I had time I would try more combinations.

Researcher: What do you mean by ‘combinations’?

Chris: Between the way these two kind of turns change. I could make it even more harmonic or complicated. (...) By using only one variable that makes each command change according to its values. The rhythm remains the same. At the three main points which are 0, 180 and 360 that the song verse changes, it is at a horizontal position; same as before. (...) If I had time I would add a variable here, instead of 2 and try and find the best relationship between them.

Chris made transitions from the digital resource to the abstract mathematics context and back in order to describe and explain the way that the dynamic figural motion was generated through his procedure. He started generalizing the joint variability of these two different variables—outputs of the functions “roll_right:y” and “right 2*:y.” He also discriminated the mathematical role of a parameter from that of a variable by suggesting the idea of using a variable to “try and find the best” dynamic condition. Time limitations did not allow Chris to continue with his exploration and shaping of meanings around variability that would maybe have led him to more transitions between MaLT2 and abstract mathematics, providing opportunities for more abstract levels of generalization.

## Discussion

The modified UDGS model turned out to be a supportive tool in order to capture students’ meaning-making process while interacting with the digital resource. The widening and further conceptualization of the using and synthesizing phases as transitions within and beyond MaLT2 respectively provided sustainable ground for describing the nature of mathematical meanings connected to music and dance. Despite the limited data of this study, its theoretical model contributed to a concrete, articulated exposition of the different forms and levels of students’ meaning-making process within the transdisciplinary and multi-representational context of the task. As indicated in the results, the diversity of representational systems reinforced the cultivation of meanings on the mathematical concepts in use. Out theoretical analysis also pointed out the role of both the digital tools and the artistic components to this process.

Four concrete flows of transitions emerged from students’ own activity, each one corresponding to one main artistic idea and the mathematical concepts around it. The artistic ideas involved the dynamic and figural aspect of their dancing animation, which was either intended to be an imitation of a real dancing move or something from their own fantasy. In this case, they used and gradually, to diverse levels, generalized mathematical concepts connected to the multidisciplinary notions of periodicity, harmony, and symmetry, such as period, circle, turn, proportional and inverse proportional relationship, variable, function, and covariation of quantities. Their engagement also involved the dynamic aspect of synchronizing their animation to the musical rhythm, where students used mathematical concepts connected to periodicity and synchronicity, such as proportional and inverse proportional relationship.

The role of the variation tool was catalytic for the exploration of such dynamic concepts and the formation of mathematical meanings around them. In fact, some students’ interaction with the variation tool was richer compared to the use of programming for making their animation and expressing their ideas, as the feedback gained from using the sliders was more direct. For example, as seen in their flow of transitions analyzed in subsections A and C, Mary and Nikos interacted exclusively with the variation tool within MaLT2 for achieving their goals. They generalized meanings around angle, input values of a variable, periodicity, and proportional and inverse proportional relationship between concrete quantities that helped them construct their envisioned dancing animation.

On the other hand, programming was connected to higher, more reflective, levels of generalization of mathematical meanings and, at the same time, more artistically spectacular creation. For example, as described in subsections B and D, even though students’ changes of the procedures coding in the editor seemed relatively minor, they signified a highly reflective way of using the generalized mathematical concepts such as periodicity, variable, and covariation that led them to the refinement of impressive animated figural dancing moves.

Throughout the process, transitions within MaLT2 were influencing transitions beyond and vice versa, highlighting their joint important role to the progression of students’ meaning-making process. In all analyzed cases, the figural and dynamic digital representations incorporating mathematical concepts in use, which were sometimes unanticipated, worked as inspiration for the formation and extension of artistic ideas. Students used mathematical concepts and relations, by transitioning within MaLT2 representations, at first in an intuitive, vague way and gradually in a more conscious, concrete one, in order to test their ideas.

These concepts, though initially implicitly embedded within the engagement with the digital tools, progressively got discriminated and generalized in order to be promptly used in a practical, meaningful way within the digital resource. The synthesizing phase, in the widened conceptualized sense of our framework, was central for all the analyzed flows of transitions. The artistic external context provided a reference point, where mathematical relations represented and explored in the digital tools could be given an additional meaningful form of application. It also provided a strong motivational boost for students’ flows of transitions that shaped the nature of the mathematical meanings and guided their overall meaning-making process.

It may be interesting to consider this research as contributing to the question of what mathematical concepts, structures, and connections may present dense interesting fields for meaning-making within Kaput’s virtual culture or rather enabled by tools, norms, and practices therein. Wilensky and Papert (2010) used the term “restructurations” to discuss readdressing what mathematics can now become fertile grounds for meaning-making given digital media. In this perspective, mathematical meanings originating from each flow of transition can be viewed as part of a novel mathematics curriculum structure, transformed by digital and programming affordances, and guided by open, creative engagement with artistic ideas around music and dance. Even though the range of this research is quite small, it provides an insightful glance at some main conceptual axes around which students were led, by using the digital tools of MaLT2 and following their own agency. This study allowed us to consider the notion of time, connecting mathematics with temporal issues and perceiving synchronicity as a potentially fertile field to generate meanings around periodicity, periodic functions, covariation, and proportionality.

## Data Availability

The transcribed data of audio recordings and interviews have been decoded so that there are no data on student identity and they are stored in secured servers of the NKUA. Parts of them can be provided after a request and a justification of use to the authors. The data are currently available in the Greek language, but parts can be translated if necessary.

## Footnotes

1 Pseudonyms were used instead of students’ real names.

2 Each song was trimmed using an online audio trimmer application. Each extract’s duration was shown next to the title of the song. They included three or more repetitive rhythmical units (verses) each.

3 As the factor of the rate of change of dragging a slider was playing an important role for this activity, it was agreed that the moving of the sliders would occur through the left and right arrows from the keyboard in order to be within a stabilized rhythm.

4 Students tended to refer to procedure inputs as “variables” instead of “parameters.”.
